# The value of double balloon enteroscopy in diagnosing blue rubber bleb naevus syndrome: a case report

**DOI:** 10.1186/1757-1626-3-29

**Published:** 2010-01-18

**Authors:** Fardod O'Kelly, Kheng Tian Lim, Narayanasamy Ravi, Nasir Mahmud, John V Reynolds

**Affiliations:** 1 Department of Clinical Surgery, Trinity Centre, St James's Hospital, Dublin 8, Ireland; 2 Department of Clinical Medicine, Trinity Centre, St James's Hospital, Dublin 8, Ireland

## Abstract

Blue rubber bleb naevus syndrome is a rare vascular disorder associated with multiple gastrointestinal haemangiomas that have the potential for life-threatening haemorrhage. These may be difficult to diagnose, and have classically been described using computed tomographic studies and/or mesenteric angiography. Resected surgical specimens of these lesions, especially in the small bowel, have often been extensive and poorly localized. The recent advent and progressive development of double balloon enteroscopy has allowed the direct visualization and marking of these enteric lesions and serves as a valuable adjunct not only in diagnosis but also planning prior to surgery to allow accurate estimate of the extent of resection.

## Introduction

Blue rubber bleb naevus syndrome, also known as Bean Syndrome was first described in 1958 on the basis of 1-2 cm violaceous cutaneous lesions, is a rare disorder consisting of gastrointestinal vascular malformations, which carry a significant potential for serious haemorrhage. The majority of cases are sporadic, however it has been postulated that this syndrome may manifest as a familial vascular malformation with autosomal dominance and associated with the TEK tyrosine kinase receptor which is almost exclusively endothelial and involved in endothelial-smooth muscle cell signaling [1]. Symptoms and signs depend on the organ system involved, ranging from none to anaemia to overt blood loss which may be massive. Patients may also complain of bone/joint pain, haemoptysis, haematuria or even blindness.

The predominant location of these lesions in the small intestine can make them difficult to diagnose. Double balloon enteroscopy was developed in 2001 as a technique to endoscopically visualize the small bowel in real time and allow therapeutic intervention such as biopsy, dilatation and placement of stents, and it has largely supplanted CT enteroclysis and angiography in the diagnosis of small bowel sources of blood loss [2].

## Case presentation

**Figure 1:**
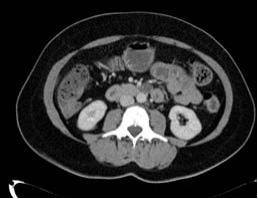
**CT enteroclysis demonstrating nodular thickening of the jejunum**.

**Figure 2:**
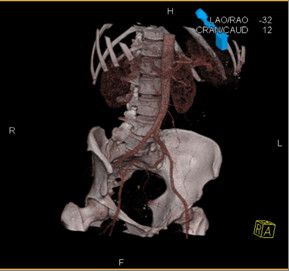
**CT mesenteric angiogram demonstrating normal enhancement of intra-abdominal vessels with no active bleeding points but evidence of small vascular anomalies**.

**Figure 3:**
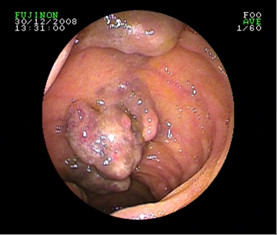
**DBE demonstrating presence of multiple areas of vascular abnormalities within the small bowel lumen**.

A 58-year-old female, Irish, Caucasian patient presented, on a background history of chronic anaemia, with fatigue, palpitations, dyspnoea and haemoglobin of 5.4 g/dL. She had received intermittent blood transfusions during the previous three years. Oesophagogastroduodenoscopy and colonoscopy were normal. She was positive for occult blood. Computed tomography (CT) enteroclysis was performed which demonstrated nodular thickening of the distal jejunum (Figure 1). CT mesenteric angiogram demonstrated multiple subcentimeter hyperenhancing foci identified throughout the jejunum with normal enhancement of the aorta and mesenteric vessels (Figure 2). The patient underwent antegrade double-balloon enteroscopy (DBE) and multiple hypervascular polypoid lesions were found resembling bunches of grapes and extending over a section of 30-40 cm in the mid-jejunum (Figure 3). The distal margin of this segment was tattood.

The patient underwent a mini laparotomy via a 5 cm incision, with segmental jejunal resection and primary hand-sewn anastomosis. She made an uneventful post-operative recovery. Histology demonstrated sections of jejunal polyps showing multiple cavernous haemangiomas, many of which showed organizing thrombus. Given the multiplicity, these were consistent with the blue rubber bleb naevus syndrome.

## Discussion

Blue rubber bleb naevus syndromes affecting the gastrointestinal tract are uncommon and, present a diagnostic challenge to those investigating cases of occult gastrointestinal anaemia or haemorrhage. There have been a number of medical modalities employed to treat this condition using anti-angiogenic agents such as corticosteroids, interferon-alpha and octreotide, but to little clinical effect [3,4]. Some have cautioned against surgery, predicting that recurrence would occur from naevi in other parts of the gastrointestinal tract [5]. Conversely, in one of few prospective studies on blue rubber bleb naevus syndrome, Fishman *et al *demonstrated the surgical resection resulted in elimination of bleeding in nine out of ten patients without recurrence over a five year follow-up period [6].

In this report, we highlight the value of DBE in pointing the location and extent of this condition. In a retrospective study by Lin *et al*, using DBE to identify gastrointestinal blood loss, localization of bleeding was provided in 95% (53/56) patients, and allowed prompt and accurate surgical resection in all but a single patient [7]. DBE appears to be a safe, useful and important adjunct prior to surgical resection of small bowel lesions including blue rubber bleb naevus syndrome, as it not only allows the precise localization and marking of these lesions, to ensure accurate resection as demonstrated in the above case, but it can also provide pre-operative histological diagnosis. By employing DBE that allows access to those areas of bowel previously only accessible by exploratory laparoscopy or laparotomy, surgical morbidity may also be reduced.

## Conclusion

DBE is a valuable adjunct not only in diagnosis but also planning prior to surgery to allow accurate estimate of the extent of resection.

## Consent

Written informed consent was obtained from the patient for publication of this case report and any accompanying images. A copy of the written consent is available for review by the Editor-in-Chief of this journal.

## Competing interests

The authors declare that they have no competing interests.

## Authors' contributions

FOK and KTL drafted the manuscript and prepared the figures. NR, NM and JVR reviewed and amended the manuscript. All authors read and approved the final manuscript.
